# Supplementary material for: Femtosecond-Laser-Pulse Characterization and Optimization for CARS Microscopy
Source: PLoS One. 2016 May 25;11(5):e0156371. doi: 10.1371/journal.pone.0156371 (PMC4880195; doi:10.1371/journal.pone.0156371)
Supplement: S1 Table — (DOCX) [file pone.0156371.s004.docx]

| ** |  | |
| --- | --- | --- |
| ** |  | |
| ** |  | |
| ** |  | |
| ** |  | |
| ** |  | |
| ** |  | |
| ** |  | |
| ** |  |  |
| ** |  |  |
