# Supplementary material for: Femtosecond-Laser-Pulse Characterization and Optimization for CARS Microscopy
Source: PLoS One. 2016 May 25;11(5):e0156371. doi: 10.1371/journal.pone.0156371 (PMC4880195; doi:10.1371/journal.pone.0156371)
Supplement: S2 Table — Errors represent the standard deviation calculated over 20 repetition of the acquisition. (DOCX) [file pone.0156371.s005.docx]

| $L_{pump}$(cm) | 0 | 0 | 10 | 10 |
| --- | --- | --- | --- | --- |
| $L_{Stokes}$ (cm) | 0 | 15 | 15 | 25 |
| $\tau_{\text{out,pump}}$(fs) | 244 ± 6 | 280 ± 25 | 520 ± 17 | 545 ± 35 |
| $\tau_{\text{out,Stokes}}$(fs) | 125 ± 10 | 435 ± 15 | 515 ± 20 | 810 ± 14 |
| $\tau_{pump}$(fs) | 155 ± 5 | 170 ± 25 | 153 ± 6 | 150 ± 12 |
| $\tau_{Stokes}$(fs) | 120 ± 10 | 120 ± 3 | 113 ± 3 | 113 ± 3 |
| $a_{pump}$(fs^2^) | 10500 ± 600 | 13700 ± 2280 | 30000 ± 2100 | 28600 ± 4000 |
| $a_{Stokes}$(fs^2^) | 1500 ± 300 | 17900 ± 1000 | 18000 ± 1000 | 32700 ± 1200 |
