# Supplementary material for: Femtosecond-Laser-Pulse Characterization and Optimization for CARS Microscopy
Source: PLoS One. 2016 May 25;11(5):e0156371. doi: 10.1371/journal.pone.0156371 (PMC4880195; doi:10.1371/journal.pone.0156371)
Supplement: S1 Text — (DOCX) [file pone.0156371.s006.docx]

## Contribution to the spectral phase of the third-order dispersion term

The group velocity dispersion (second-order dispersion) and the third-order dispersion (TOD) introduced by a block of glass with wavelength-dependent refractive index $n(\lambda)$ can be calculated as:

,

.

Using the Sellmeier approximation for the refractive index of SF6 reported in the article, it is possible to estimate GVD and TOD introduced by such glass numerically at *λ*_0_ = 810 nm:

GVD ≈ 196000 fs^2^/m;

TOD ≈ 125000 fs^3^/m.

Considering a laser with 100 fs pulses, i.e. with a spectral bandwidth$\delta\omega\approx2.8\times10^{13} \text{s}^{-1}$, the contribution to the spectral phase of the GVD and TOD for a 10-cm SF6 block can be estimated as:

$GVD\cdot\delta\omega^{2}/2\approx7.54$;

$TOD\cdot\delta\omega^{3}/6\approx0.04$,

the latter being about 2% of the linear term.
